# High amphipathicity of α-helical peptides enhances unmethylated CpG DNA-induced activation of mouse macrophage-like RAW264.7 cells

**DOI:** 10.1038/s41598-024-67166-8

**Published:** 2024-07-15

**Authors:** Saeka Nishihara, Nao Nakamura, Kiyoshi Kawasaki

**Affiliations:** Faculty of Pharmaceutical Sciences, Doshisha Women’s College of Liberal Arts, Kyotanabe, Kyoto 610-0395 Japan

**Keywords:** Innate immunity, Antimicrobial peptide, Toll-like receptor 9, Helical wheel projection, Cellular uptake of DNA, Biochemistry, Cell biology, Immunology

## Abstract

The α-helical antimicrobial peptide Kn2-7 enhances the activation of mouse macrophage-like RAW264.7 induced by DNA containing unmethylated cytosine-guanine motifs (CpG DNA). This enhancement is related to increased cellular uptake of DNA by Kn2-7, but the relevant properties of Kn2-7 are unknown. Physicochemical property analysis revealed that Kn2-7 has high amphipathicity. In contrast, the α-helical antimicrobial peptide L5, which increases the cellular uptake of CpG DNA but does not enhance CpG DNA-induced activation, has low amphipathicity. Kn2-7 derivatives with decreased amphipathicity but the same amino acid composition as Kn2-7 did not enhance CpG DNA-induced activation. On the other hand, L5 derivatives with high amphipathicity but the same amino acid composition as L5 enhanced CpG DNA-induced activation. Cellular uptake of DNA was not increased by the L5 derivatives, indicating that high amphipathicity does not affect DNA uptake. Furthermore, α-helical peptides with reversed sequences relative to the Kn2-7 and L5 derivatives with high amphipathicity were synthesized. The reversed-sequence peptides, which had the same amphipathicity but different amino acid sequences from their counterparts, enhanced CpG DNA-induced activation. Taken together, these observations indicate that the high amphipathicity of α-helical peptides enhances the CpG DNA-induced activation of RAW264.7.

## Introduction

In mammals, pathogen-associated molecular patterns, such as lipopolysaccharide (LPS), flagellin, lipopeptide, and DNA containing unmethylated cytosine-guanine motifs (CpG DNA), are recognized by innate immune receptors, including Toll-like receptors (TLRs)^1^. This recognition initiates innate immune responses to eliminate the invading pathogens, such as the induction of inflammatory responses, leading to the activation of adaptive immunity^1^. TLR9, which is localized in endosomes and lysosomes, recognizes endocytosed CpG DNA that is more prevalent in bacteria than vertebrates^2,3^. CpG DNA induces not only inflammatory responses, including secretion of pro-inflammatory cytokines, but also the activation of the T helper 1-biased adaptive immune response and the proliferation of B cells^4–6^. Therefore, CpG DNA has been clinically used to treat illnesses such as infectious diseases and immune-related diseases, and also as a vaccine adjuvant^4–6^.

We have focused on antimicrobial peptides (AMPs) as agents that enhance CpG DNA-induced activation of immune cells. AMPs are important bactericidal effectors of innate immunity in many species, ranging from plants and insects to vertebrates^7^. The diversity of AMPs is so great that they are broadly categorized based on their secondary structure, such as α-helical peptides and β-sheet peptides^8,9^. Clustering of cationic and hydrophobic amino acids into distinct domains is observed in several antimicrobial peptides of different structural classes. This amphipathic structure is evident in many, but not all, antimicrobial peptides^10^. A large family of AMPs assumes an amphipathic α-helical structure upon contact with cell membranes^10–12^. These physicochemical properties permit AMPs to bind to microbial membranes through electrostatic interactions with the negatively charged microbial surface molecules, such as LPS, and to insert into the membrane via hydrophobic interactions, leading to increased membrane permeability and disruption^10–12^. Furthermore, some AMPs can modulate immune responses initiated by TLR activation^13^. This ability depends on the positive charge, hydrophobicity, and secondary structure of AMPs, since these characteristics are important for the interaction of these AMPs with negatively charged TLR ligands such as LPS, CpG DNA, and polyinosinic-polycytidylic acid (Poly(I:C)), the last of which mimics viral double-stranded RNA^13^. For instance, some cationic AMPs, including the amphipathic α-helical peptides CP29 and CEMA, can directly bind to LPS and inhibit LPS-induced cytokine secretion from macrophages^14^. Also, the human amphipathic α-helical peptide LL-37 enhances Poly(I:C)-induced TLR3 signaling^15^.

Kn2-7 (FIKRIARLLRKIF) is an α-helical antimicrobial peptide derived from the venom of scorpion *Buthus martensii* Karsch^16,17^. Recently, we found that Kn2-7 increased CpG DNA-induced cytokine secretion from mouse macrophage-like RAW264.7 cells^18^. We also showed that Kn2-7 increased the cellular uptake of CpG DNA, and this ability was related to the enhancement of CpG DNA-induced activation of RAW264.7 cells^18^. However, the CpG DNA-induced response was not enhanced by Kn2-7 synthesized with D-amino acids, which can increase the cellular uptake of CpG DNA^18^. Furthermore, the α-helical antimicrobial peptide L5 (KLKLLLLLKLK)^19,20^, which increases the cellular uptake of CpG DNA^21^, did not enhance the CpG DNA-induced activation of RAW264.7 cells^18^. Therefore, the increased responsiveness to CpG DNA caused by these peptides is assumed to be due not only to increased cellular uptake of CpG DNA, but also to other effects resulting from these peptides’ physicochemical properties. In this study, we investigated the physicochemical properties of peptides involved in the enhancement of CpG DNA-induced activation of RAW264.7 cells. We focused on the amphipathicity of α-helical peptides and found that high amphipathicity enhanced this activation.

## Results

### Kn2-7 exhibits high amphipathicity, and L5 exhibits low amphipathicity

**Figure 1:**
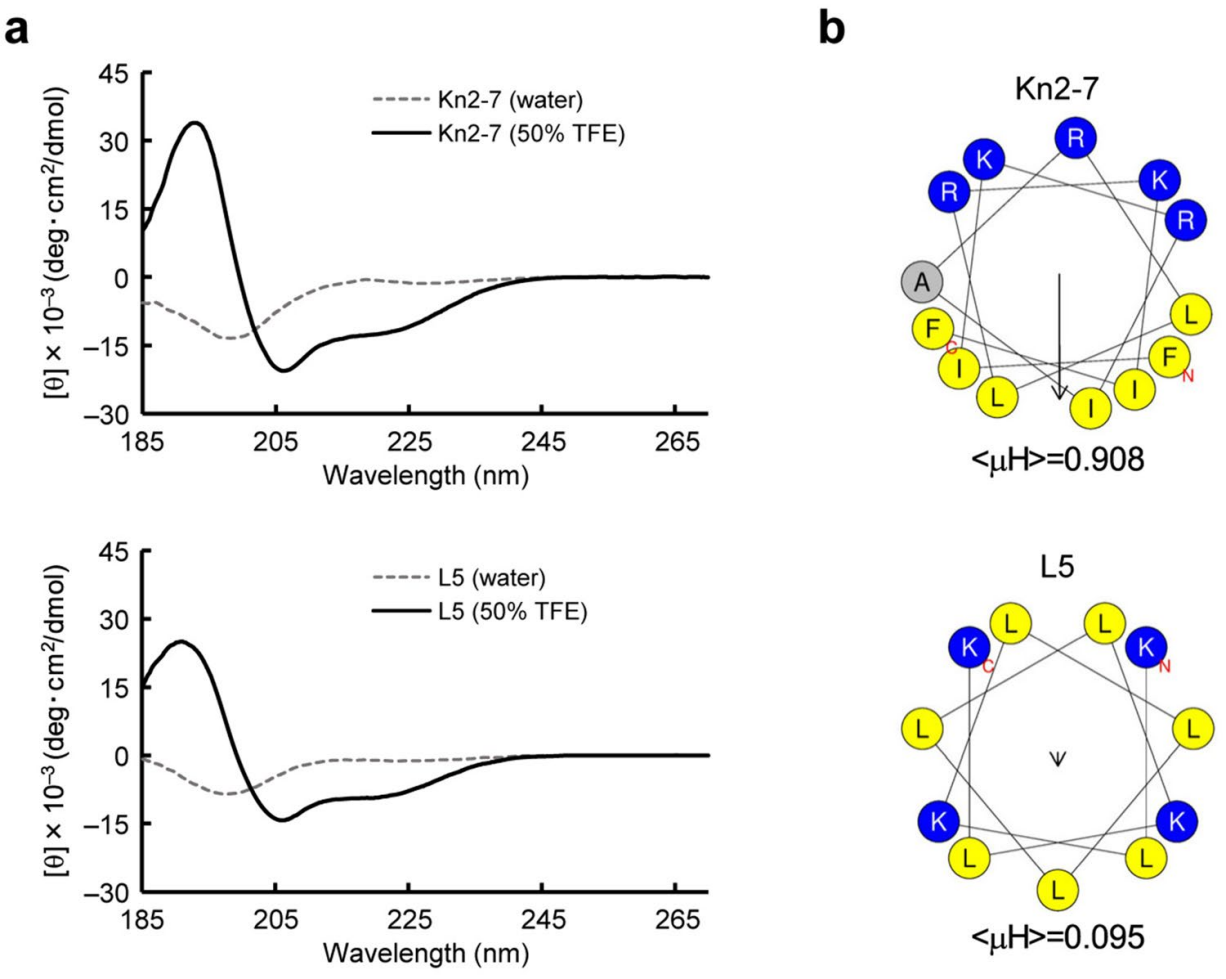
Characterization of α-helical properties of Kn2-7 and L5. (**a**) CD spectra of Kn2-7 (upper panel) and L5 (lower panel) were measured in 50% TFE (solid black line) or water (dotted gray line), at a concentration of 0.1 mg/ml. The mean residue molar ellipticity ([θ], deg･cm^2^/dmol) was determined, and the value of [θ] × 10^‒3^ was plotted against the wavelength. (**b**) The helical wheel projection and mean hydrophobic moment, < μH > , for Kn2-7 and L5 were obtained using HeliQuest. Positively charged amino acids are represented by blue circles, hydrophobic residues by yellow circles, and the alanine residue by a gray circle. The arrow in the projections indicates the magnitude and direction of < μH > for each peptide, with the corresponding < μH > value shown below each projection.

To explore the physicochemical properties of Kn2-7 that are involved in the enhancement of CpG DNA-induced activation of RAW264.7 cells, we compared Kn2-7, which enhances this activation, with L5, which does not^18^. Since both Kn2-7 and L5 are known to form an α-helical structure^17,22^, we used the web server HeliQuest to calculate the physicochemical properties of α-helical peptides to obtain the helical wheel projection and the mean hydrophobic moment, < μH > (Fig. 1)^23^. According to the helical wheel projection, Kn2-7 exhibits a typical, highly amphipathic structure: one face of the helix contains mainly hydrophilic residues, while the opposite face consists mainly of hydrophobic residues (Fig. 1b). In contrast, the helical wheel projection of L5 does not exhibit amphipathicity (Fig. 1b). The amphipathicity of α-helical peptides is reflected by the < μH > value, with a high value indicating high amphipathicity^24^. According to a HeliQuest prediction, the < μH > values of Kn2-7 and L5 were 0.908 and 0.095, respectively (Fig. 1b). Since high amphipathicity is defined as a < μH > value higher than 0.6^23^, the amphipathicity of Kn2-7 is high and that of L5 is low.

### Kn2-7 derivatives with decreased amphipathicity do not enhance CpG DNA-induced activation of RAW264.7 cells

**Figure 2:**
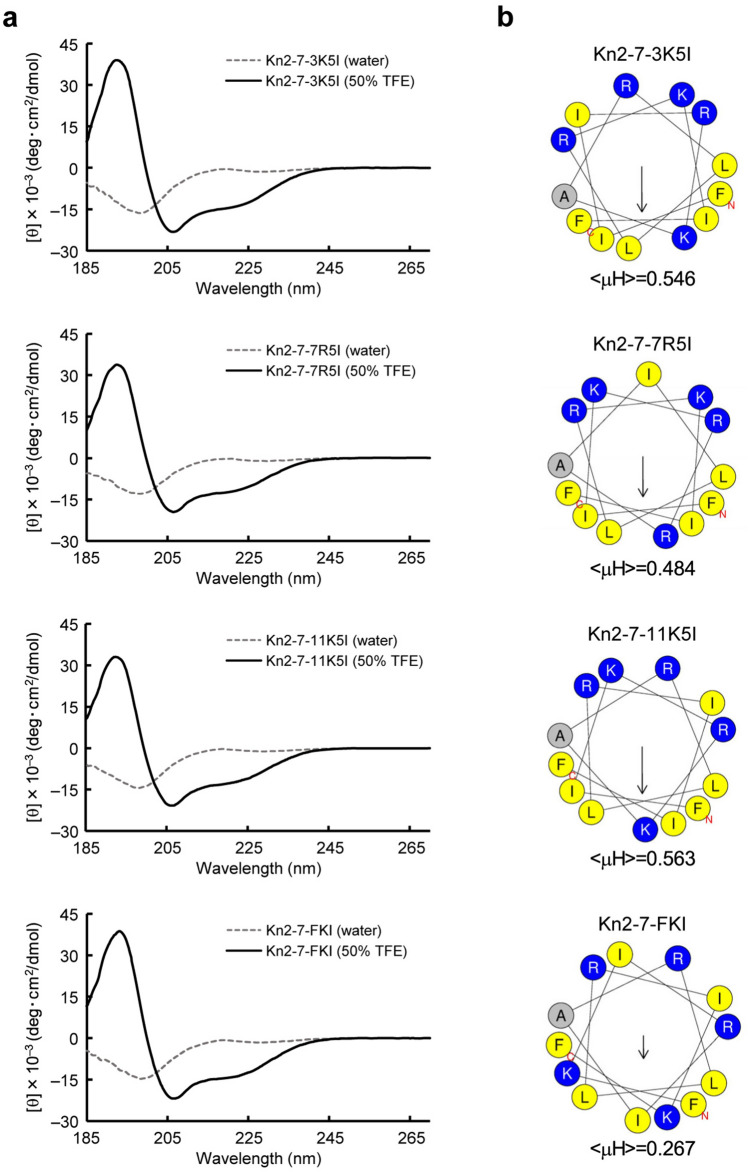
Characterization of α-helical properties of Kn2-7 derivatives. (**a**) CD spectra of the Kn2-7 derivatives Kn2-7-3K5I, Kn2-7-7R5I, Kn2-7-11K5I, and Kn2-7-FKI (from top to bottom) were measured in 50% TFE (solid black line) or water (dotted gray line), at a concentration of 0.1 mg/ml. The mean residue molar ellipticity ([θ], deg･cm^2^/dmol) was determined, and the value of [θ] × 10^‒3^ was plotted against the wavelength. (**b**) The helical wheel projection and mean hydrophobic moment, < μH > , for each Kn2-7 derivative shown in (**a**) were obtained using HeliQuest. Positively charged amino acids are represented by blue circles, hydrophobic residues by yellow circles, and alanine residues by gray circles. The arrow in the projections indicates the magnitude and direction of < μH > for each peptide, with the corresponding < μH > value shown below each projection.

To analyze the involvement of the high amphipathicity of Kn2-7 in enhancing CpG DNA-induced activation of RAW264.7 cells, we designed Kn2-7 derivatives with lower amphipathicity than Kn2-7 but with the same amino acid composition. We selectively exchanged one or two hydrophilic residues on the polar face of Kn2-7 with hydrophobic residues on the nonpolar face. The resultant Kn2-7 derivatives were as follows: Kn2-7-3K5I, in which the third lysine was exchanged with the fifth isoleucine; Kn2-7-7R5I, in which the seventh arginine was exchanged with the fifth isoleucine; Kn2-7-11K5I, in which the 11th lysine was exchanged with the fifth isoleucine; and Kn2-7-FKI, in which the third and 11th lysines were exchanged with the second and 12th isoleucines, respectively (Table 1). To confirm the α-helical structures of these Kn2-7 derivatives, we measured the circular dichroism (CD) spectra of the peptides in 50% trifluoroethanol (TFE), which mimics membrane environments^25,26^. In 50% TFE, Kn2-7 and its derivatives exhibited a typical α-helical spectrum, with two negative peaks, at around 208 and 222 nm, and a positive peak at around 190 nm, while the α-helical spectrum was not observed without TFE (Figs. 1a and 2a). Since the Kn2-7 derivatives formed an α-helical structure in the membrane-mimetic environment, we obtained the helical wheel projections and < μH > values of the derivatives using HeliQuest. The < μH > values of Kn2-7-3K5I, Kn2-7-7R5I, Kn2-7-11K5I, and Kn2-7-FKI were 0.546, 0.484, 0.563, and 0.267, respectively (Fig. 2b). The < μH > values of the derivatives were lower than that of Kn2-7, indicating successful disruption of the amphipathic structure of Kn2-7 (Fig. 2b).

**Table 1:** Synthetic peptides used in this study.

Group	Peptide	Sequence
	Kn2-7	FIKRIARLLRKIF
Kn2-7 derivatives	Kn2-7-3K5I	FIIRKARLLRKIF
	Kn2-7-7R5I	FIKRRAILLRKIF
	Kn2-7-11K5I	FIKRKARLLRIIF
	Kn2-7-FKI	FKIRIARLLRIKF
	Kn2-7-reverse	FIKRLLRAIRKIF
	Kn2-7-3K5I-reverse	FIKRLLRAKRIIF
	Kn2-7-7R5I-reverse	FIKRLLIARRKIF
	Kn2-7-11K5I-reverse	FIIRLLRAKRKIF
	Kn2-7-FKI-reverse	FKIRLLRAIRIKF
	L5	KLKLLLLLKLK
L5 derivatives	L5-3K4L	KLLKLLLLKLK
	L5-3K8L	KLLLLLLKKLK
	L5-KLL	KLLKLLLKLLK
	L5-KLL-shift2	LKLLLKLLKKL
	L5-KLL-shift5	LLKLLKKLLKL
	L5-3K4L-reverse	KLKLLLLKLLK
	L5-3K8L-reverse	KLKKLLLLLLK
	L5-KLL-shift2-reverse	LKKLLKLLLKL
	L5-KLL-shift5-reverse	LKLLKKLLKLL

**Figure 3:**
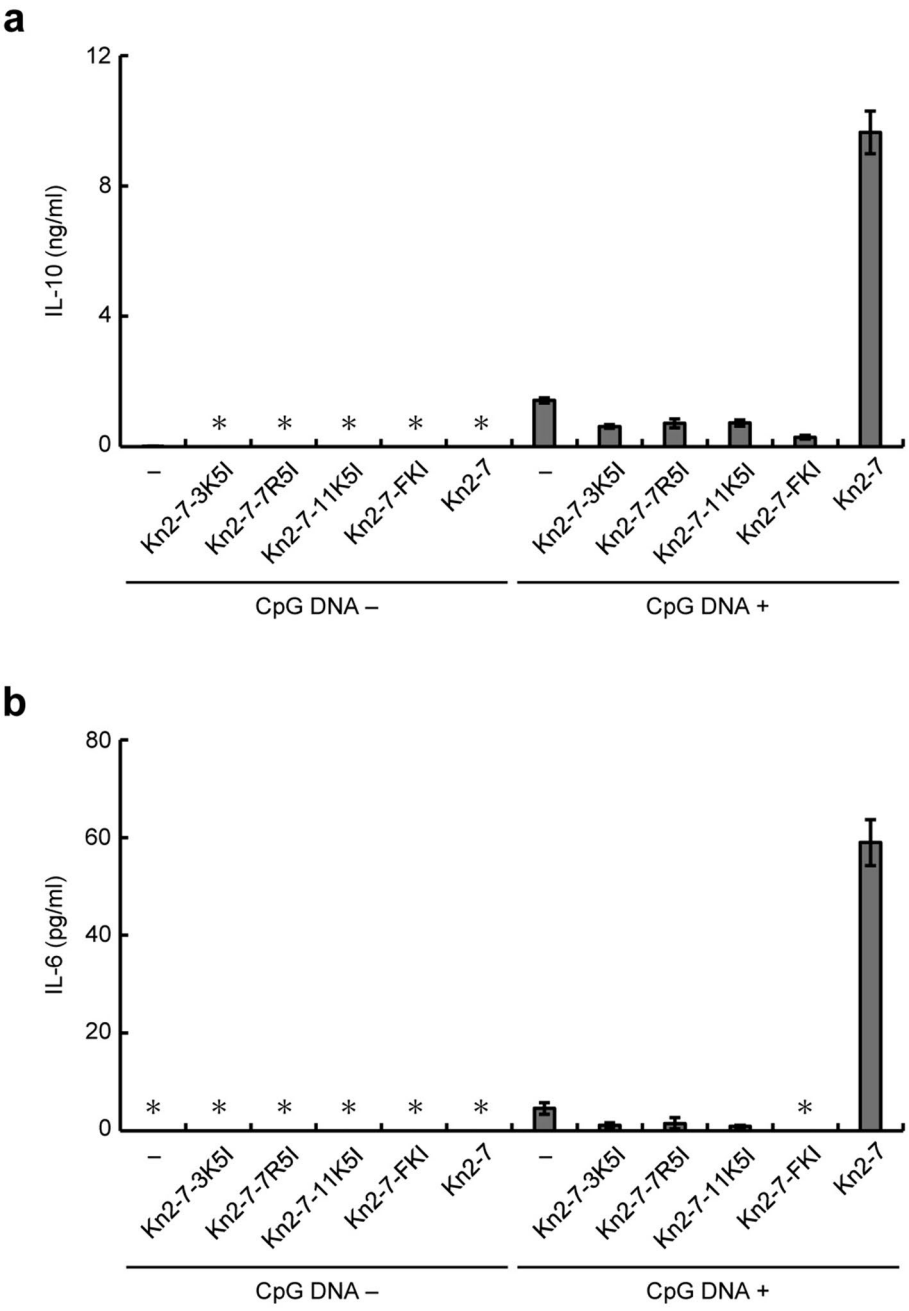
The effects of Kn2-7 derivatives with decreased amphipathicity on CpG DNA-induced activation of RAW264.7 cells. RAW264.7 cells were stimulated with (CpG DNA +) or without (CpG DNA −) CpG DNA in the absence (−) or presence of Kn2-7-3K5I, Kn2-7-7R5I, Kn2-7-11K5I, Kn2-7-FKI, or Kn2-7 at a concentration of 10 μg/ml. Concentrations of IL-10 (**a**) and IL-6 (**b**) in culture supernatants are expressed as means ± standard deviations from triplicate wells. Asterisks (*) indicate that IL-10 and IL-6 were not detected (below blank levels).

We next examined the effects of these derivatives on the CpG DNA-induced activation of RAW264.7 cells by monitoring the secretion of interleukin (IL)-10 and IL-6. We have previously demonstrated that Kn2-7 enhances CpG DNA-dependent secretion of IL-10 and tumor necrosis factor-α (TNF-α)^18^. In this study, we monitored IL-6 instead of TNF-α because the enhancement of IL-6 is clearer than that of TNF-α (Supplementary Fig. 1). In the presence of Kn2-7 (10 μg/ml), the secretion of IL-10 and IL-6 from RAW264.7 cells in response to CpG DNA stimulation was approximately 6.8- and 13-fold higher, respectively, compared with that in the absence of Kn2-7 (Fig. 3a,b). In contrast, the CpG DNA-dependent secretion of IL-10 and IL-6 was not increased in the presence of Kn2-7-3K5I, Kn2-7-7R5I, Kn2-7-11K5I, or Kn2-7-FKI, each at a concentration of 10 μg/ml, compared with the absence of these derivatives (Fig. 3a,b). These results, taken together, indicated that Kn2-7 derivatives with decreased amphipathicity do not enhance the CpG DNA-induced activation of RAW264.7 cells.

### L5 derivatives with high amphipathicity enhance the CpG DNA-induced activation of RAW264.7 cells

**Figure 4:**
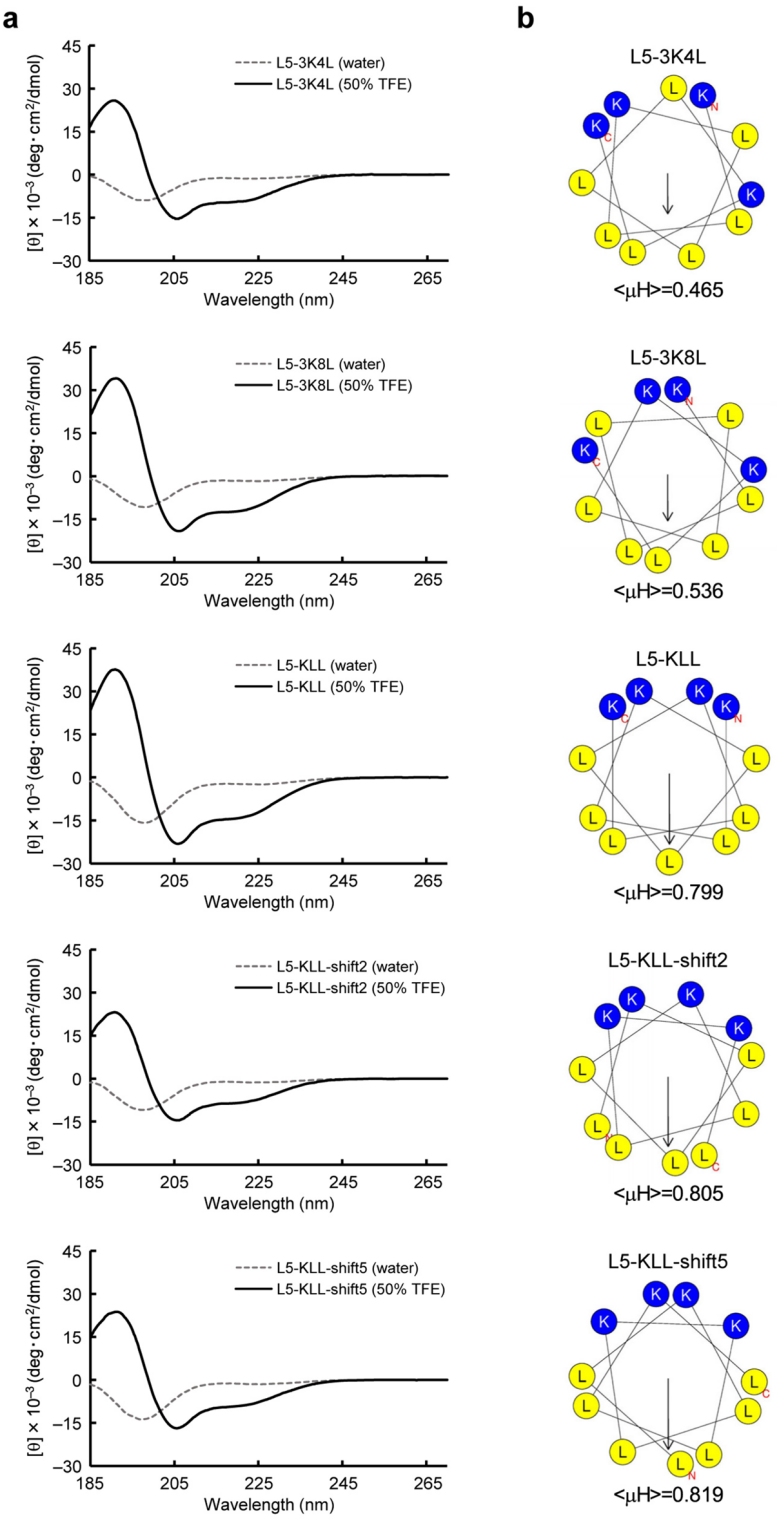
Characterization of α-helical properties of L5 derivatives. (**a**) CD spectra of the L5 derivatives L5-3K4L, L5-3K8L, L5-KLL, L5-KLL-shift2, and L5-KLL-shift5 (from top to bottom) were measured in 50% TFE (solid black line) or water (dotted gray line), at a concentration of 0.1 mg/ml. The mean residue molar ellipticity ([θ], deg cm^2^/dmol) was determined, and the value of [θ] × 10^‒3^ was plotted against the wavelength. (**b**) The helical wheel projection and mean hydrophobic moment, < μH > , for each L5 derivative shown in (**a**) were obtained using HeliQuest. Positively charged amino acids are represented by blue circles, and hydrophobic residues by yellow circles. The arrow in the projections indicates the magnitude and direction of < μH > for each peptide, with the corresponding < μH > value shown below each projection.

We also designed L5 derivatives with higher amphipathicity than L5 but with the same amino acid composition, to determine if the high amphipathicity of α-helical peptides enhances the CpG DNA-induced activation of RAW264.7 cells. We selectively exchanged one or two hydrophilic L5 residues with hydrophobic residues, converging the hydrophilic residues onto one face and the hydrophobic residues onto the opposite face. The resultant L5 derivatives were as follows: L5-3K4L, in which the third lysine was exchanged with the fourth leucine; L5-3K8L, in which the third lysine was exchanged with the eighth leucine; and L5-KLL, in which the third and ninth lysines were exchanged with the fourth and eighth leucines, respectively (Table 1). To confirm the α-helical structures of these L5 derivatives, we measured the CD spectra of the peptides. L5-3K4L, L5-3K8L, and L5-KLL, like L5, each exhibited a typical α-helical spectrum in 50% TFE (Figs. 1a and 4a). Since L5-3K4L, L5-3K8L, and L5-KLL formed an α-helical structure in the membrane-mimetic environment, we obtained the helical wheel projection and < μH > value of each derivative using HeliQuest. The < μH > values of L5-3K4L, L5-3K8L, and L5-KLL were 0.465, 0.536, and 0.799, respectively (Fig. 4b), indicating that L5-KLL has high amphipathicity and that L5-3K4L and L5-3K8L have intermediate amphipathicity. Furthermore, we designed highly amphipathic L5 derivatives in which the L5-KLL sequence was sequentially shifted one residue at a time in the N-terminal direction. Two resultant peptides, L5-KLL-shift2 (shifted by two residues) and L5-KLL-shift5 (shifted by five residues) (Table 1), were selected because their < μH > values were higher than that of L5-KLL (Fig. 4b). Both peptides formed an α-helical structure in 50% TFE by CD measurement (Fig. 4a).

**Figure 5:**
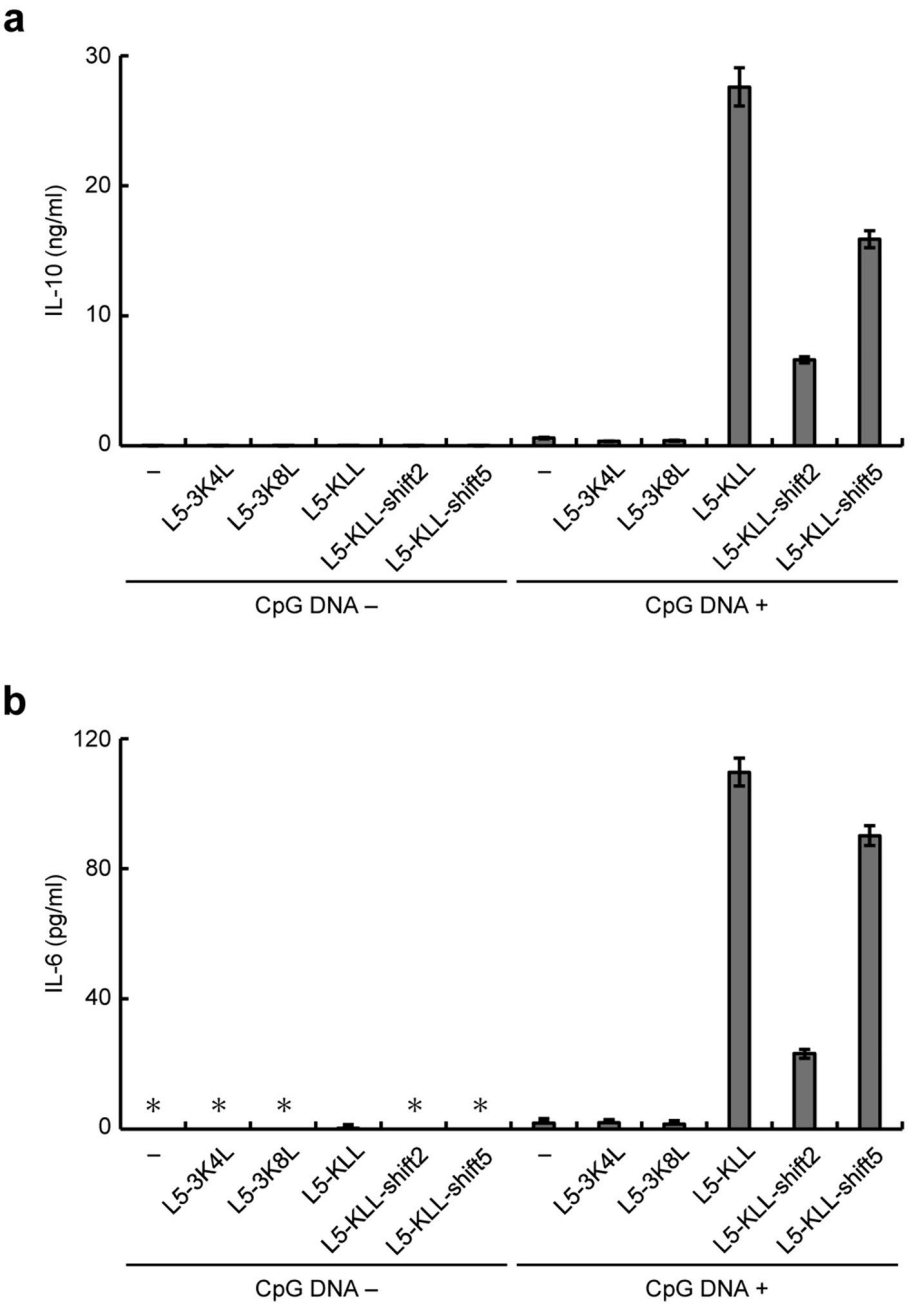
The effects of L5 derivatives with increased amphipathicity on CpG DNA-induced activation of RAW264.7 cells. RAW264.7 cells were stimulated with (CpG DNA +) or without (CpG DNA −) CpG DNA in the absence (−) or presence of L5-3K4L, L5-3K8L, L5-KLL, L5-KLL-shift2, or L5-KLL-shift5 at a concentration of 10 μg/ml. Concentrations of IL-10 (**a**) and IL-6 (**b**) in culture supernatants are expressed as means ± standard deviations from triplicate wells. Asterisks (*) indicate that IL-6 was not detected (below blank levels).

We next examined the effects of the L5 derivatives on the secretion of IL-10 and IL-6 from RAW264.7 cells stimulated with CpG DNA. As shown in Fig. 5a, CpG DNA-dependent IL-10 secretion was approximately 46.2-, 11.1-, and 26.6-fold higher in the presence of 10 μg/ml of L5-KLL, L5-KLL-shift2, and L5-KLL-shift5, respectively, compared to that in the absence of these derivatives. Similarly, CpG DNA-dependent IL-6 secretion was approximately 63.3-, 13.3-, and 52.0-fold higher in the presence of 10 μg/ml of L5-KLL, L5-KLL-shift2, and L5-KLL-shift5, respectively, compared to that in the absence of these derivatives (Fig. 5b). The secretion of IL-10 and IL-6 was not induced by these derivatives in the absence of CpG DNA (Fig. 5). There was a dose-dependent increase in CpG DNA-dependent IL-6 secretion by L5-KLL, L5-KLL-shift2, and L5-KLL-shift5 (Supplementary Fig. 2). These observations indicated that L5-KLL, L5-KLL-shift2, and L5-KLL-shift5 enhanced the CpG DNA-induced activation of RAW264.7 cells. In contrast, in the presence of 10 μg/ml of L5-3K4L or L5-3K8L, the secretion of IL-10 and IL-6 in response to CpG DNA stimulation was similar to that in the absence of these derivatives (Fig. 5a,b). Taken together, L5 derivatives with high amphipathicity enhance the CpG DNA-induced activation of RAW264.7 cells.

### L5 derivatives with high amphipathicity do not increase the cellular uptake of CpG DNA by RAW264.7 cells

The ability of Kn2-7 to increase the cellular uptake of CpG DNA was previously shown to be related to the enhancement of CpG DNA-induced activation of RAW264.7 cells^18^. The punctate pattern of fluorescence-labeled CpG DNA in the cytoplasm was increased in the presence of Kn2-7. The increased fluorescence of CpG DNA was co-localized with the red fluorescence of LysoTracker, indicating that the CpG DNA is localized to late endosomes and lysosomes (Supplementary Fig. 3). Furthermore, the fluorescence of LysoTracker detected by CpG DNA stimulation was increased by co-stimulation with Kn2-7, indicating that Kn2-7 enhances CpG DNA-induced activation of RAW264.7 cells (Supplementary Fig. 3).

**Figure 6:**
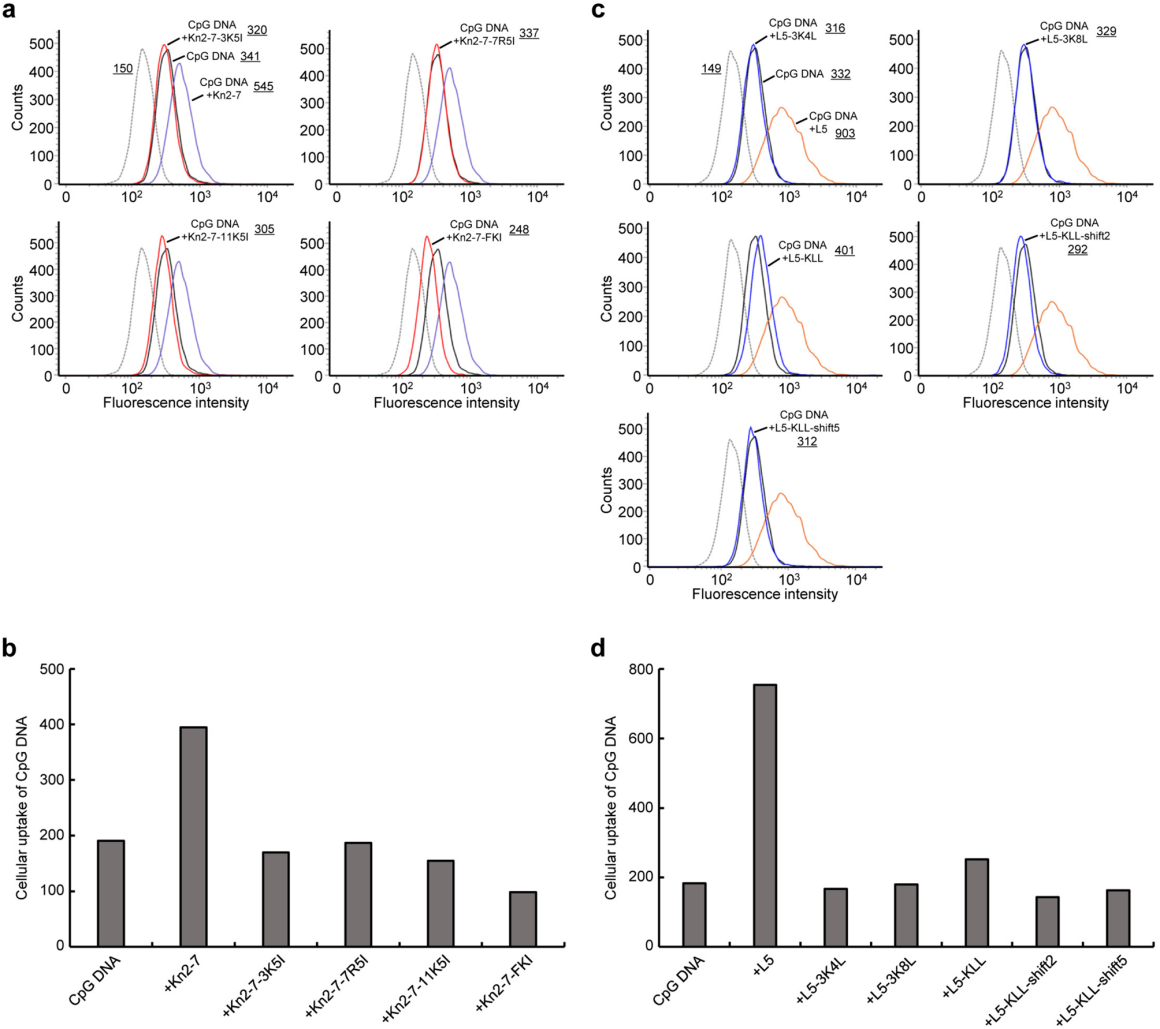
The effects of Kn2-7 derivatives and L5 derivatives on the cellular uptake of CpG DNA by RAW264.7 cells. (**a** and **c**) RAW264.7 cells were stimulated with CpG DNA-FAM alone (solid black line) or with CpG DNA-FAM in the presence of (**a**) 10 μg/ml Kn2-7 derivatives (solid red lines) or 10 μg/ml Kn2-7 (solid purple line), or (**c**) 10 μg/ml L5 derivatives (solid blue lines) or 10 μg/ml L5 (solid orange line). The fluorescence intensities of cells analyzed by flow cytometry are shown. (**a**) For clarity of comparison, the results are separated into four panels for each Kn2-7 derivative: Kn2-7-3K5I in the upper left, Kn2-7-7R5I in the upper right, Kn2-7-11K5I in the lower left, and Kn2-7-FKI in the lower right. The same histograms are presented in all panels for direct comparison: cells without stimulation (dotted gray line), cells stimulated with CpG DNA-FAM alone, and cells stimulated with CpG DNA-FAM in the presence of Kn2-7. (**c**) For clarity of comparison, the results are separated into five panels for each L5 derivative: L5-3K4L in the upper left, L5-3K8L in the upper right, L5-KLL in the center left, L5-KLL-shift2 in the center right, and L5-KLL-shift5 in the bottom. The same histograms are presented in all panels for direct comparison: cells without stimulation, cells stimulated with CpG DNA-FAM alone, and cells stimulated with CpG DNA-FAM in the presence of L5. The underlined values in histograms indicate Geo MFIs. (**b** and **d**) Cellular uptakes of CpG DNA in (**a**) and (**c**) are shown, respectively. Cellular uptake of CpG DNA was defined by subtracting the Geo MFI value of cells without CpG DNA stimulation from that of cells with CpG DNA stimulation.

We examined the effects of the Kn2-7 derivatives on the cellular uptake of CpG DNA by RAW264.7 cells. The cellular uptake of fluorescence-labeled CpG DNA was increased approximately 2.1-fold in the presence of Kn2-7 compared with the absence of Kn2-7 (Fig. 6a,b). On the other hand, in the presence of Kn2-7-3K5I, Kn2-7-7R5I, Kn2-7-11K5I, or Kn2-7-FKI, the cellular uptake of CpG DNA was not increased compared with that in the absence of these derivatives (Fig. 6a,b). These observations indicated that Kn2-7 derivatives with decreased amphipathicity did not increase the cellular uptake of CpG DNA.

We also examined the effects of L5 derivatives on the cellular uptake of CpG DNA by RAW264.7 cells. In the presence of L5-3K4L, L5-3K8L, L5-KLL, L5-KLL-shift2, or L5-KLL-shift5, the cellular uptake of CpG DNA was similar to that observed without these derivatives (Fig. 6c,d). In contrast, L5 increased CpG DNA uptake by approximately 4.1-fold compared with that without L5 (Fig. 6c,d). These observations indicated that L5 derivatives with high amphipathicity did not increase the cellular uptake of CpG DNA. Therefore, the enhancement of CpG DNA-induced activation of RAW264.7 cells by L5 derivatives with high amphipathicity was not related to increased cellular uptake of CpG DNA.

### Peptides with reversed sequences relative to Kn2-7 and L5 derivatives exhibit the same high amphipathicity but different amino acid sequences from their counterparts, enhance the CpG DNA-induced activation of RAW264.7 cells

**Figure 7:**
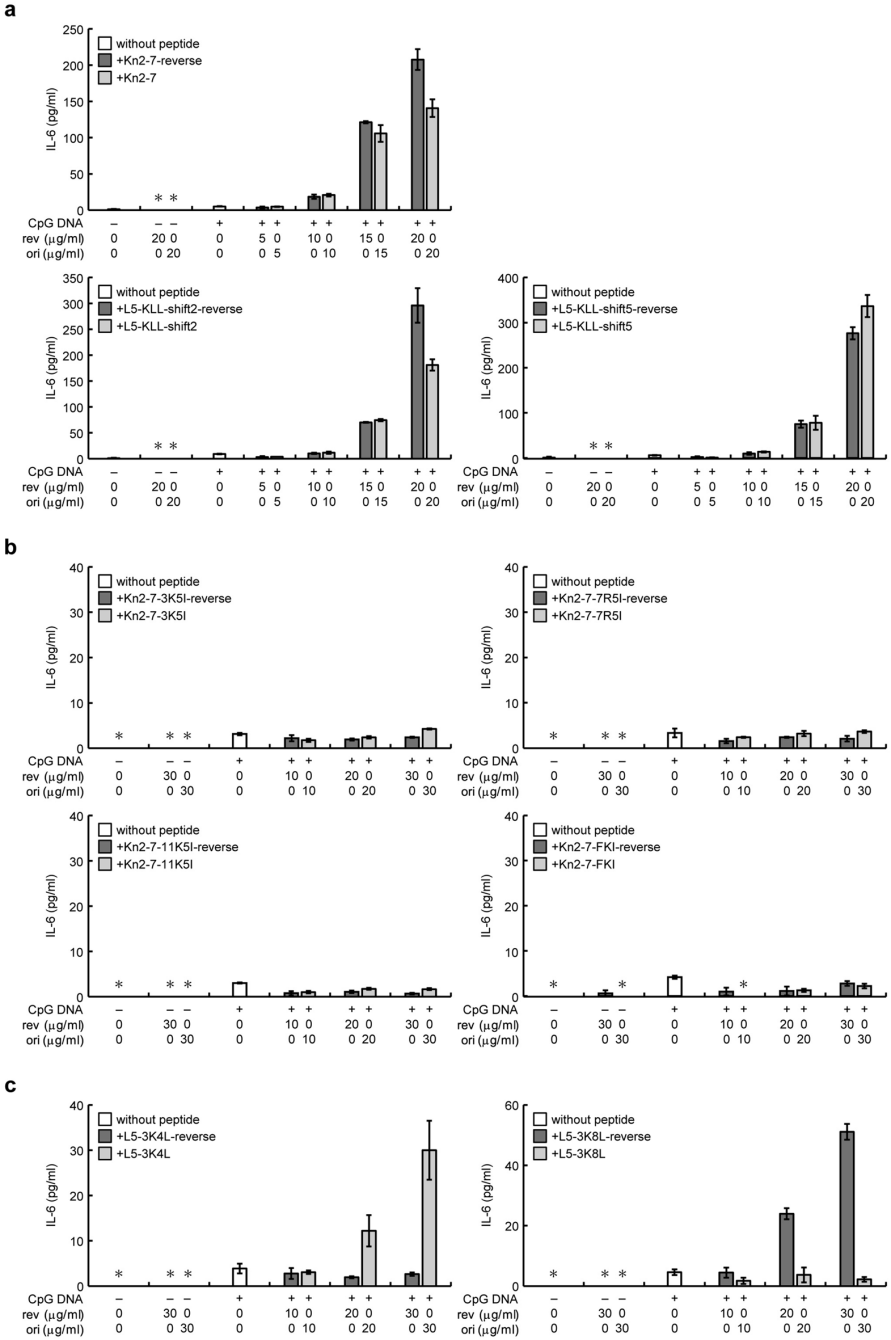
The effects of peptides with reversed sequences relative to Kn2-7, its derivatives, and L5 derivatives, on CpG DNA-induced activation of RAW264.7 cells. RAW264.7 cells were stimulated with (+) or without (−) CpG DNA in the presence of (**a**) Kn2-7-reverse and Kn2-7 (upper panel), L5-KLL-shift2-reverse and L5-KLL-shift2 (lower left panel), or L5-KLL-shift5-reverse and L5-KLL-shift5 (lower right panel); (**b**) Kn2-7-3K5I-reverse and Kn2-7-3K5I (upper left panel), Kn2-7-7R5I-reverse and Kn2-7-7R5I (upper right panel), Kn2-7-11K5I-reverse and Kn2-7-11K5I (lower left panel), or Kn2-7-FKI-reverse and Kn2-7-FKI (lower right panel); (**c**) L5-3K4L-reverse and L5-3K4L (left panel), or L5-3K8L-reverse and L5-3K8L (right panel). Peptide concentrations (μg/ml) are indicated. Concentrations of IL-6 in culture supernatants stimulated with or without CpG DNA, in the presence of the indicated reversed-sequence peptides (rev; dark gray bars) and their counterparts (ori; light gray bars), or in the absence of these peptides (white bars), are expressed as means ± standard deviations from triplicate wells. Asterisks (*) indicate that IL-6 was not detected (below blank levels).

To further investigate the importance of the high amphipathicity of α-helical peptides in the enhancement of CpG DNA-induced activation of RAW264.7 cells, we synthesized peptides whose sequences were reversed relative to the α-helical peptides used in this study (Table 1), except for L5 and L5-KLL, as their reversed sequences are the same as those of their counterparts. The < μH > values of the reversed-sequence peptides were the same as those of their counterparts (Supplementary Fig. 4), indicating no changes in amphipathicity^27^. Thus, we examined the enhancement of CpG DNA-induced activation of RAW264.7 cells using following the reversed-sequence peptides: (i) highly amphipathic peptides, specifically Kn2-7-reverse, L5-KLL-shift2-reverse, and L5-KLL-shift5-reverse, whose sequences were the reverse of Kn2-7, L5-KLL-shift2, and L5-KLL-shift5, respectively; (ii) intermediately amphipathic peptides, specifically Kn2-7-3K5I-reverse, Kn2-7-7R5I-reverse, Kn2-7-11K5I-reverse, and Kn2-7-FKI-reverse, whose sequences were the reverse of Kn2-7-3K5I, Kn2-7-7R5I, Kn2-7-11K5I, and Kn2-7-FKI, respectively, and L5-3K4L-reverse and L5-3K8L-reverse, whose sequences were the reverse of L5-3K4L and L5-3K8L, respectively. As shown in Fig. 7a, CpG DNA-dependent IL-6 secretion was increased by Kn2-7-reverse, L5-KLL-shift2-reverse, and L5-KLL-shift5-reverse in a dose-dependent manner. The increase in IL-6 secretion by these derivatives and their counterparts occurred at concentrations higher than 10 μg/ml, indicating all had a similar ability to enhance CpG DNA-induced RAW264.7 activation (Fig. 7a). Also, at concentrations of 10, 20, and 30 μg/ml, Kn2-7-3K5I-reverse, Kn2-7-7R5I-reverse, Kn2-7-11K5I-reverse, and Kn2-7-FKI-reverse, as well as their counterparts, did not increase CpG DNA-dependent IL-6 secretion (Fig. 7b). Similarly, CpG DNA-dependent IL-10 secretion was not increased in the presence of 10, 20, or 30 μg/ml of the reversed-sequence peptides or their counterparts (Supplementary Fig. 5). Taken together, these results indicate that the amphipathic structure of α-helical peptides, but not their specific amino acid sequence, is important in their ability to increase the responsiveness to CpG DNA.

Unexpectedly, however, CpG DNA-dependent IL-6 secretion was increased in the presence of 20 and 30 μg/ml L5-3K4L but not in the presence of the same concentrations of L5-3K4L-reverse (Fig. 7c; left panel). These results were consistent with the observation that 10 μg/ml L5-3K4L did not increase IL-6 secretion (Fig. 5b). Also, L5-3K8L-reverse increased CpG DNA-dependent IL-6 secretion in a dose-dependent manner, but L5-3K8L did not (Fig. 7c; right panel). These results indicate that L5-3K4L and L5-3K8L-reverse enhanced CpG DNA-dependent cellular activation, whereas their counterparts did not. Therefore, the enhancement of CpG DNA-induced activation by α-helical peptides can be attributed not only to their amphipathic structure, but also, in cases of intermediate amphipathicity, to other properties such as peptide sequence.

Furthermore, we examined the effects of peptides with reversed sequences relative to Kn2-7 and L5 derivatives on the cellular uptake of CpG DNA. As shown in Supplementary Fig. 6a, b, Kn2-7-reverse increased the cellular uptake of CpG DNA, and the level of increase was similar to that of Kn2-7. On the other hand, reversed-sequence peptides for L5 derivatives did not increase the cellular uptake of CpG DNA (Supplementary Fig. 6c,d). These observations, taken together, indicated that peptides with reversed sequences relative to Kn2-7 and L5 derivatives have the similar effect on the cellular uptake of CpG DNA as their counterparts (Fig. 6 and Supplementary Fig. 6). Moreover, the enhancement of CpG DNA-induced activation of RAW264.7 cells by reversed-sequence peptides for L5 derivatives was not related to increased cellular uptake of CpG DNA.

## Discussion

In this study, we focused on the amphipathicity of α-helical peptides to identify the properties responsible for the enhancement of CpG DNA-induced activation of RAW264.7 cells. The results were summarized in Supplementary Table 1. Our study revealed that high amphipathicity is the main contributor to this enhancement, but in cases where the amphipathicity is intermediate, peptide amino acid sequences may play a significant role. While the specific sequence properties of α-helical peptides that enhance CpG DNA-induced activation have not been identified, some properties shared between L5-3K4L and L5-3K8L-reverse may play a role, in addition to their amphipathicity.

We also analyzed the relationship between the amphipathicity of α-helical peptides and their cellular uptake of CpG DNA. We found that Kn2-7 derivatives with decreased amphipathicity did not increase the cellular uptake of CpG DNA by RAW264.7 in comparison with Kn2-7. These observations were consistent with our previous study, in which we showed that increased cellular uptake of CpG DNA was related to the Kn2-7-dependent enhancement of CpG DNA-induced activation of RAW264.7 cells^18^. Therefore, the inability of Kn2-7 derivatives to enhance CpG DNA-induced activation of RAW264.7 cells can be attributed to their failure to increase cellular uptake of CpG DNA. On the other hand, L5 derivatives and their reversed-sequence peptides with high amphipathicity did not increase RAW264.7 cells’ uptake of CpG DNA but did increase their responsiveness to it. Therefore, the fact that α-helical peptides increase the responsiveness of these cells to CpG DNA is unrelated to these peptides’ ability to increase cellular CpG DNA uptake. The amphipathic structure of α-helical peptides, including Kn2-7 and L5 derivatives with high amphipathicity, is likely to contribute to the enhancement of CpG DNA-induced activation of RAW264.7 cells.

It has been reported that the structure and size of DNA fragments are important factors influencing immune responses. For instance, Y-shaped oligodeoxynucleotides (ODNs) containing CpG motifs were prepared using three ODNs with the halves of each ODN being partially complementary to a half of the other two ODNs, and they were found to induce greater secretion of TNF-α and IL-6 from RAW264.7 cells than conventional single- or double-stranded ODNs containing identical numbers of CpG motifs^28^. In another study utilizing polypod-like DNA structures containing CpG motifs, DNA nano-assemblies composed of three to eight pods were synthesized and investigated in terms of the relationships between their structural and immunological properties^29^. The results demonstrated that higher numbers of pods increased the secretion of TNF-α and IL-6 from RAW264.7 cells, irrespective of the number of nucleotides in one polypod-like DNA structure^29^. The binding of CpG DNA to TLR9 triggers receptor dimerization and initiates cellular signaling^30^. More recently, it was shown that in terms of maximizing TLR9 activation and immune response, the optimal distance between CpG binding domains within the TLR9 dimer is 7 nm^31,32^. These studies, taken together, indicate that not only the number of CpG motifs but also CpG spatial organization contribute to the magnitude of TLR9 signaling. Previously, we showed that Kn2-7 and L5 each bind to CpG DNA, forming large complexes^21^. The binding of CpG DNA to α-helical peptides with highly amphiphilic structure should be crucial for the CpG spatial organization to enhance the CpG DNA-dependent immune responses. Although we did not determine the mechanisms by which the Kn2-7 and L5 derivatives with high amphipathicity increased the CpG DNA-induced responsiveness of RAW264.7 cells, these peptides might enhance TLR9 activation by influencing the spatial organization of CpG motifs.

Furthermore, it has been reported that TAT peptide, a typical cell-penetrating peptide (CPP), is functionalized to dendrimer-like CpG DNA nanostructures for enhanced cellular uptake and immune responses^33^. CPPs are short peptides comprising fewer than 35 amino acids, and can traverse the cellular membrane, delivering biologically active molecules such as oligonucleotides, proteins, and nanoparticles^34^. CPPs can be divided into three main classes, namely cationic, hydrophobic, and amphipathic^34^, and some of their characteristics are shared with those of AMPs^35^. In our study, Kn2-7 and L5 may function as CPPs because they bind to CpG DNA and deliver it to endosomes^18,21^, and thus their derivatives may also behave as CPPs, even though their ability to increase cellular CpG DNA uptake was much reduced compared to Kn2-7 and L5. The observation that L5 derivatives with high amphipathicity enhanced CpG DNA-induced activation of RAW264.7 cells suggests that the intracellular localization of uptaken CpG DNA is much more important than the amount of this uptake in terms of enhancing CpG DNA-induced cell activation. The entrapment of CpG DNA in endosomes might positively influence CpG DNA-dependent immune responses, because TLR9 activation is initiated in endosomes^3^. Therefore, L5 derivatives with high amphipathicity might favor the accumulation of CpG DNA inside endosomes. Further analysis of peptide properties, including the structure of their complexes with CpG DNA and their intracellular localization, is required to reveal the molecular mechanisms by which highly amphipathic α-helical peptides enhance the CpG DNA-induced activation of immune cells.

## Materials and methods

### DNAs and peptides

CpG DNA was purchased from InvivoGen (CA, USA). The sequence was 5′-TCC ATG ACG TTC CTG ATG CT-3′ (ODN 1668), and all bases contained a phosphorothioate backbone. CpG DNA labeled with fluorescein at the 3′ terminus (CpG DNA-FAM) was synthesized by Nippon Gene (Toyama, Japan).

Peptides listed in Table 1 were commercially synthesized by Scrum Incorporated (Tokyo, Japan). The purity of the synthesized peptides was > 90%. The C-termini of the peptides were modified by amidation.

### Analysis of peptides’ secondary structure

Peptides’ secondary structure was analyzed by CD measurements. Peptides (0.1 mg/ml) were dissolved in water containing 50% (vol/vol) TFE or water, and CD spectra were measured at 25℃ by a J-1500 spectrometer (JASCO, Tokyo, Japan) using a quartz cuvette with a 2-mm path length. The spectra were recorded from 270 to 185 nm with a bandwidth of 1 nm at a scanning speed of 100 nm/min, and data were collected every 0.2 nm and accumulated 10 times. All spectra were smoothed with a convolution width of 9 using the Savitzky–Golay method^36^.

### HeliQuest analysis of α-helical peptides

The helical wheel projection and the mean hydrophobic moment, < μH > , of the α-helical peptides were obtained using the HeliQuest online program (http://heliquest.ipmc.cnrs.fr)^23^.

### Cell culture

The mouse macrophage-like cell line RAW264.7 (DS Pharma Biomedical, Osaka, Japan) was cultivated in high-glucose Dulbecco’s modified Eagle’s medium (Sigma-Aldrich, MO, USA) supplemented with 10% (vol/vol) heat-inactivated (56 ℃ for 30 min) fetal bovine serum (Sigma-Aldrich), 100 units/ml penicillin (Thermo Fisher Scientific, MA, USA), and 100 μg/ml streptomycin (Thermo Fisher Scientific) at 37 ℃ in a humidified environment with 5% CO_2_.

### Analysis of cytokine secretion by sandwich enzyme-linked immunosorbent assay (ELISA)

RAW264.7 cells (5 × 10^5^ cells/ml) were seeded on 48-well microtiter plates (Corning Incorporated, NY, USA) at 400 μl/well, and the cells were cultivated overnight. After washing once with 400 μl of the culture medium, the cells were stimulated with 10 nM CpG DNA in the presence or absence of the indicated concentrations of peptides for 24 h. Then, the culture supernatants were collected and the concentrations of IL-10 and IL-6 were measured by sandwich ELISA using a Mouse IL-10 Uncoated ELISA Kit and a Mouse IL-6 Uncoated ELISA Kit (Invitrogen Life Technologies, CA, USA), respectively.

### Analysis of cellular uptake of CpG DNA by flow cytometry

RAW264.7 cells (5 × 10^5^ cells/ml) were seeded on 12-well microtiter plates (Sumitomo Bakelite, Tokyo, Japan) at 1.2 ml/well, and the cells were cultivated overnight. After washing once with 1.2 ml of the culture medium, the cells were stimulated with 50 nM CpG DNA-FAM in the presence or absence of the peptide (each at 10 μg/ml) for 2 h. Then, the cells were washed three times with 1.2 ml of ice-cold phosphate-buffered saline (PBS; 137 mM NaCl, 2.7 mM KCl, 1.47 mM KH_2_PO_4_, 8.1 mM Na_2_HPO_4_), and were suspended in 2.4 ml of ice-cold PBS. Forward and side scatter were used to gate on cells, and a region of large, healthy cells was defined using unstimulated cells. Ten thousand cells in the region were analyzed using a FACSLyric flow cytometer (Becton Dickinson Biosciences, CA, USA). The sampling rate was medium (60 μl/min). The excitation/emission wavelengths were 488/511–543 nm. Data analysis was performed using FACSuite software (Becton Dickinson Biosciences), and the geometric mean of fluorescence intensity (Geo MFI) was determined.

### Supplementary Information

Supplementary Information.

## Data Availability

All data generated or analyzed during this study are included in the published article and its supplementary information file.
