# Supplementary material for: High amphipathicity of α-helical peptides enhances unmethylated CpG DNA-induced activation of mouse macrophage-like RAW264.7 cells
Source: Sci Rep. 2024 Jul 15;14:16274. doi: 10.1038/s41598-024-67166-8 (PMC11251158; doi:10.1038/s41598-024-67166-8)
Supplement: Supplementary file 1 — Supplementary Information. [file 41598_2024_67166_MOESM1_ESM.pdf]

Supplementary information

High amphipathicity of α-helical peptides enhances unmethylated CpG DNA-induced activation of mouse macrophage-like RAW264.7 cells

Saeka Nishihara, Nao Nakamura, and Kiyoshi Kawasaki*
Faculty of Pharmaceutical Sciences, Doshisha Women's College of Liberal Arts, Kyotanabe, Kyoto 610-0395, Japan.

*Corresponding author, E-mail address: kkawasak@dwc.doshisha.ac.jp

Supplementary Figure 1-6
Supplementary Table 1

Supplementary Figure 1

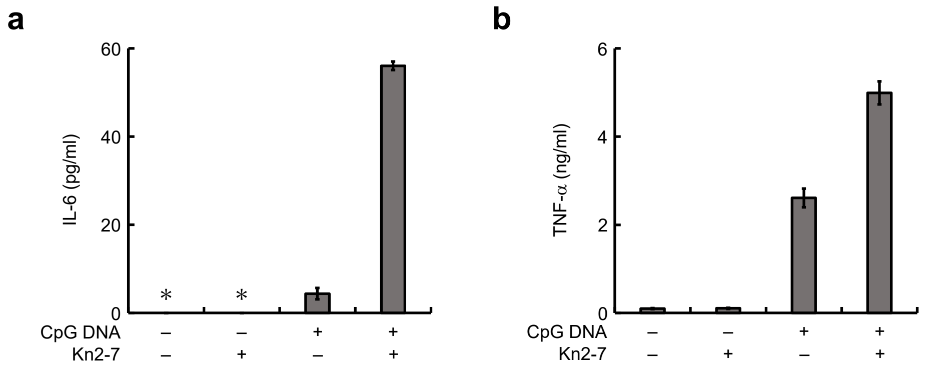

Supplementary Figure 1. The comparison of Kn2-7 effects on CpG DNA-dependent secretion of IL-6 and TNF-α from RAW264.7 cells.

RAW264.7 cells were stimulated with (+) or without (–) CpG DNA in the presence (+) or absence (–) of 10 μg/ml Kn2-7. Concentrations of IL-6 (**a**) and TNF-α (**b**) in culture supernatants are expressed as means ± standard deviations from triplicate wells. Asterisks (*) indicate that IL-6 was not detected (below blank levels).

Supplementary Figure 2

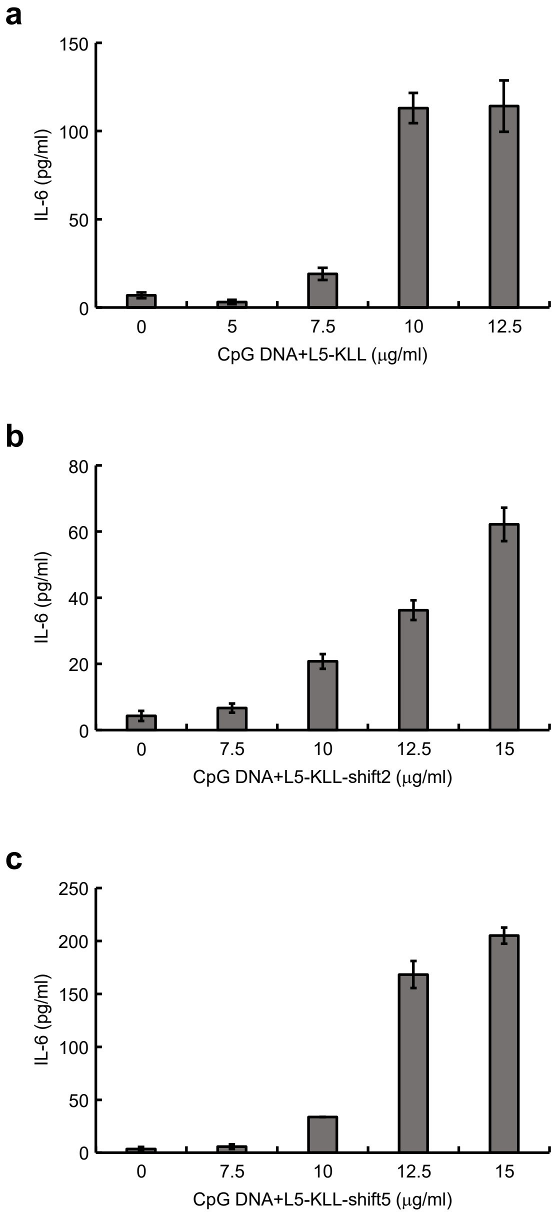

Supplementary Figure 2. Dose-dependent effects of L5-KLL, L5-KLL-shift2, and L5-KLL-shift5 on CpG DNA-dependent IL-6 secretion from RAW264.7 cells.

RAW264.7 cells were stimulated with CpG DNA in the presence of L5-KLL (**a**), L5-KLL-shift2 (**b**), or L5-KLL-shift5 (**c**). Peptide concentrations (μg/ml) are indicated. Concentrations of IL-6 in culture supernatants are expressed as means ± standard deviations from triplicate wells.

Supplementary Figure 3

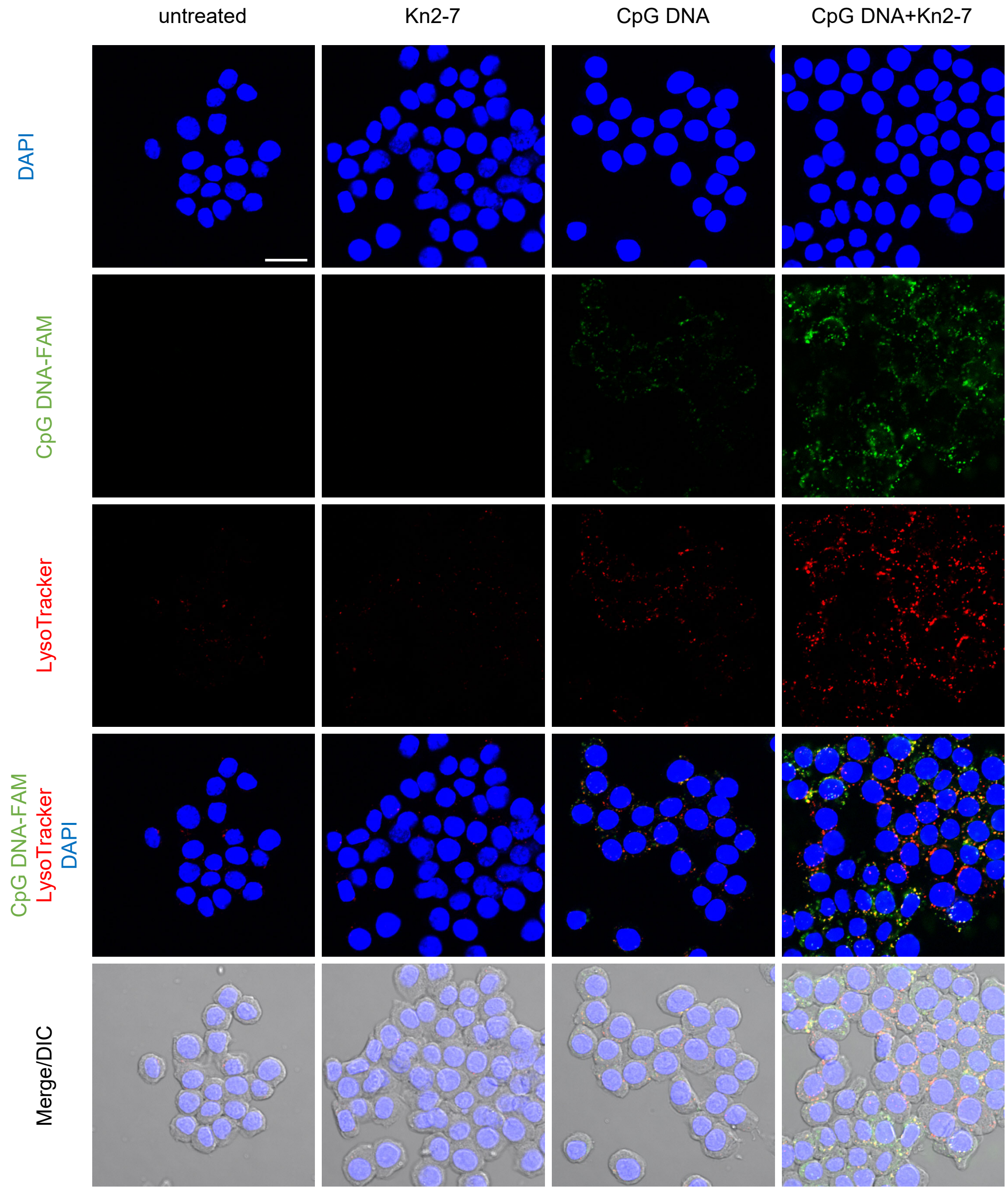

Supplementary Figure 3. Analysis of cellular localization of internalized CpG DNA.
RAW264.7 cells were stimulated with or without CpG DNA-FAM in the presence or absence of 7.5 µg/ml Kn2-7. Subsequently, acidic organelles such as late endosomes and lysosomes were stained with LysoTracker, and the cells were analyzed by confocal microscopy. Nuclei stained with 4’,6-diamidino-2-phenylindole (DAPI, blue), CpG DNA-FAM (green), LysoTracker (red), merged images, and merged images with differential interference contrast (DIC) are shown. The scale bar indicates 20 µm.

The method of confocal microscopy was performed as described previously with slight modifications (S. Nishihara and K. Kawasaki, *Biochem. Biophys. Res. Commun.*, 530, 100–106, 2020). Briefly, RAW264.7 cells were cultivated overnight. After washing once with culture medium, the cells were stimulated with or without 50 nM CpG DNA-FAM in the presence or absence of 7.5 µg/ml Kn2-7 for 2 h. Then, the cells were washed three times with culture medium, and treated with 400 µl of 1 µM LysoTracker Red DND-99 (Life Technologies) for 30 min under cell culture conditions. After washing three times with PBS, the cells were fixed and the nuclei were stained with DAPI. Subsequently, the mounted cells were analyzed using a Nikon A1R confocal laser scanning microscope equipped with a 10× eyepiece, a 40× objective lens, and an electronic 3× zoom. Excitation/emission wavelengths were 405/425–475 nm (for DAPI), 488/500–550 nm (for FAM), and 561/570–620 nm (for LysoTracker).

Supplementary Figure 4

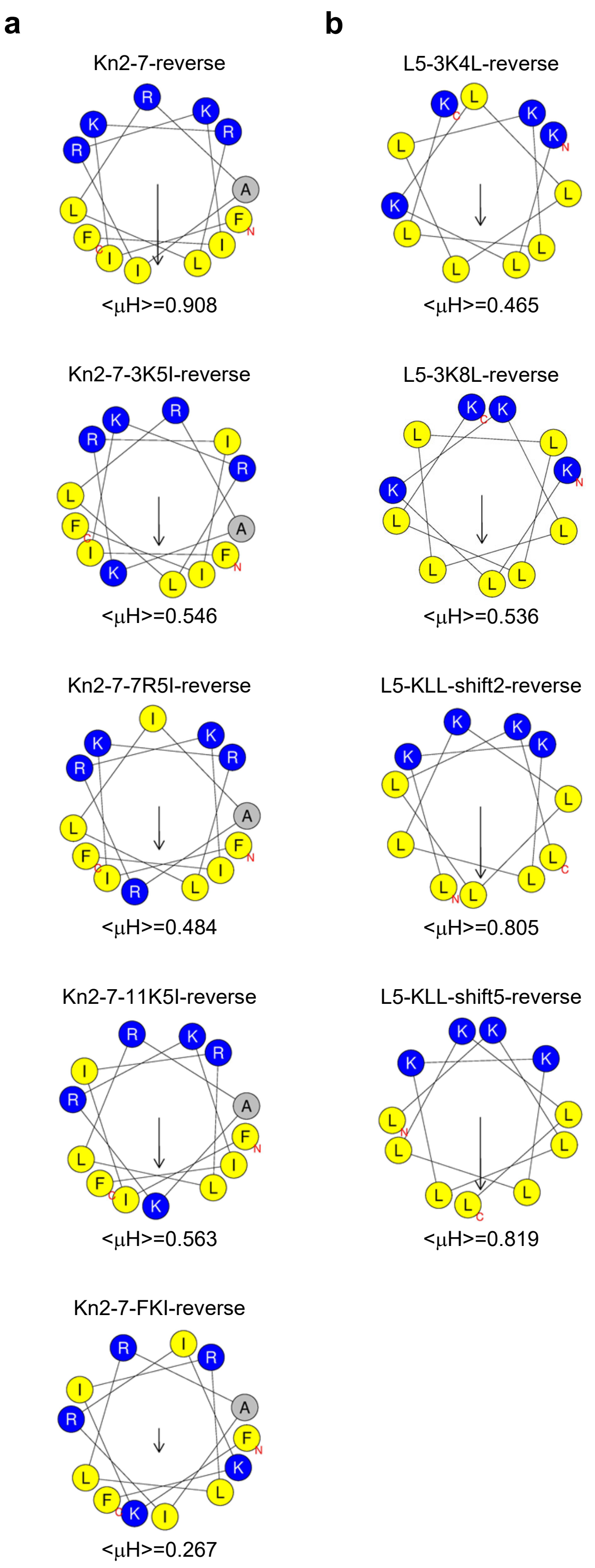

Supplementary Figure 4. HeliQuest analysis of peptides with reversed sequences relative to Kn2-7, its derivatives, and L5 derivatives.

Helical wheel projections and mean hydrophobic moments, <μH>, for peptides with reversed sequences relative to Kn2-7, its derivatives, and L5 derivatives were obtained using HeliQuest. The reversed-sequence peptides for Kn2-7 and its derivatives are shown in (**a**): from top to bottom, Kn2-7-reverse, Kn2-7-3K5I-reverse, Kn2-7-7R5I-reverse, Kn2-7-11K5I-reverse, and Kn2-7-FKI-reverse. The reversed-sequence peptides for L5 derivatives are shown in (**b**): from top to bottom, L5-3K4L-reverse, L5-3K8L-reverse, L5-KLL-shift2-reverse, and L5-KLL-shift5-reverse. Positively charged amino acids are represented by blue circles, hydrophobic residues by yellow circles, and alanine residues by gray circles. The arrow in the projections indicates the magnitude and direction of <μH> for each peptide, with the corresponding <μH> value shown below each projection.

Supplementary Figure 5

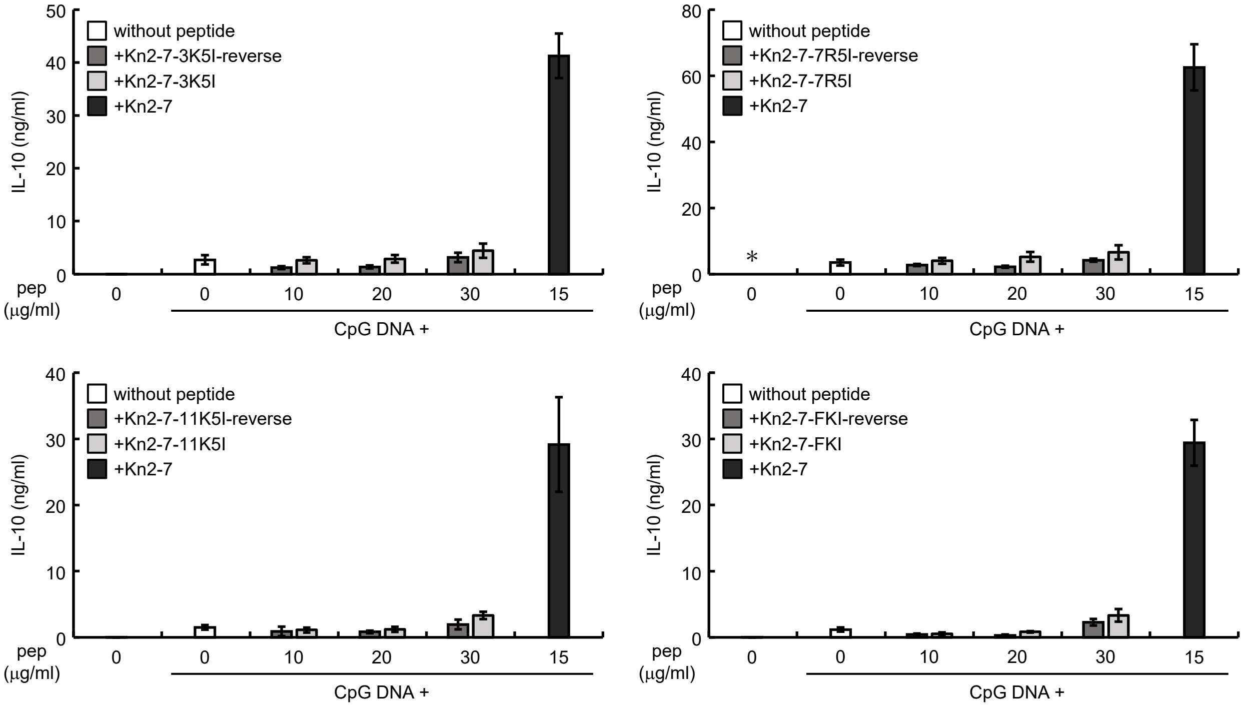

Supplementary Figure 5. The effects of peptides with reversed sequences relative to Kn2-7 derivatives on CpG DNA-dependent IL-10 secretion from RAW264.7 cells.

RAW264.7 cells were stimulated with (CpG DNA +) or without CpG DNA in the presence of Kn2-7-3K5I-reverse and Kn2-7-3K5I (upper left panel), Kn2-7-7R5I-reverse and Kn2-7-7R5I (upper right panel), Kn2-7-11K5I-reverse and Kn2-7-11K5I (lower left panel), or Kn2-7-FKI-reverse and Kn2-7-FKI (lower right panel). Peptide (pep) concentrations (μg/ml) are indicated. Concentrations of IL-10 in culture supernatants stimulated with CpG DNA, in the presence of the indicated reversed-sequence peptides (dark gray bars), their counterparts (light gray bars), and 15 μg/ml Kn2-7 (black bars), or in the absence of these peptides (white bar), are expressed as means ± standard deviations from triplicate wells. The asterisk (*) indicates that IL-10 was not detected (below blank levels).

Supplementary Figure 6

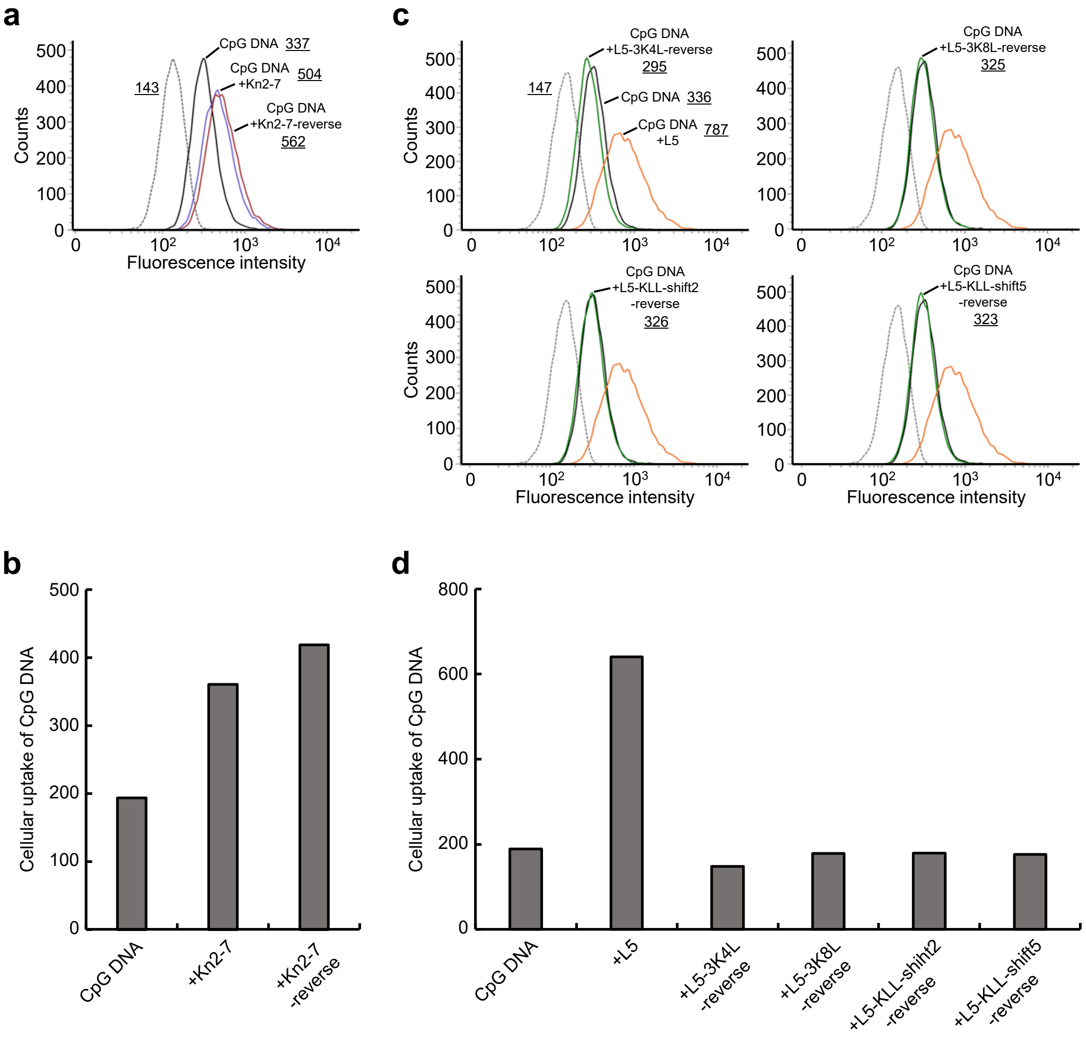

Supplementary Figure 6. The effects of peptides with reversed sequences relative to Kn2-7 and L5 derivatives on the cellular uptake of CpG DNA by RAW264.7 cells.

(**a** and **c**) RAW264.7 cells were stimulated with CpG DNA-FAM alone (solid black line) or with CpG DNA-FAM in the presence of (**a**) 10 μg/ml Kn2-7-reverse (solid brown line) or 10 μg/ml Kn2-7 (solid purple line), or (**c**) 10 μg/ml reversed-sequence peptides for L5 derivatives (solid green lines) or 10 μg/ml L5 (solid orange line). The fluorescence intensities of cells analyzed by flow cytometry are shown. (**c**) For clarity of comparison, the results are separated into four panels for each reversed-sequence peptides for L5 derivatives: L5-3K4L-reverse in the upper left, L5-3K8L-reverse in the upper right, L5-KLL-shift2-reverse in the lower left, and L5-KLL-shift5-reverse in the lower right. The same histograms are presented in all panels for direct comparison: cells without stimulation (dotted gray line), cells stimulated with CpG DNA-FAM alone, and cells stimulated with CpG DNA-FAM in the presence of L5. The underlined values in histograms indicate Geo MFIs. (**b** and **d**) Cellular uptakes of CpG DNA in (**a**) and (**c**) are shown, respectively. Cellular uptake of CpG DNA was defined by subtracting the Geo MFI value of cells without CpG DNA stimulation from that of cells with CpG DNA stimulation.

Supplementary Table 1

Group	peptide	<μH>	original	reversed
	Kn2-7	0.908	○	○
Kn2-7 derivatives	Kn2-7-3K5I	0.546	—	—
	Kn2-7-7R5I	0.484	—	—
	Kn2-7-11K5I	0.563	—	—
	Kn2-7-FKI	0.267	—	—
	L5	0.095	—	—*
L5 derivatives	L5-3K4L	0.465	△	—
	L5-3K8L	0.536	—	△
	L5-KLL	0.799	○	○*
	L5-KLL-shift2	0.805	○	○
	L5-KLL-shift5	0.819	○	○

Supplementary Table 1. Summary of the effects of peptides on the CpG DNA-induced cell activation.

Kn2-7, L5, and their derivatives are indicated as "original", and their reversed-sequence peptides are indicated as "reversed". ○ and △ indicate that the peptides enhance the CpG DNA-induced activation of RAW264.7 cells, at 10 μg/ml and at concentrations higher than 10 μg/ml, respectively. — indicates that the peptides do not enhance the activation. *The reversed sequences of L5 and L5-KLL are the same as their original peptides.
